# Overexpression of a set of genes, including WISP-1, common to pulmonary metastases of both mouse D122 Lewis lung carcinoma and B16-F10.9 melanoma cell lines

**DOI:** 10.1038/sj.bjc.6600977

**Published:** 2003-07-15

**Authors:** O Margalit, L Eisenbach, N Amariglio, N Kaminski, A Harmelin, R Pfeffer, M Shohat, G Rechavi, R Berger

**Affiliations:** 1 Pediatric Hemato-Oncology, Safra Children's Hospital, Sheba Medical Center, Affiliated to Tel-Aviv University, Tel Hashomer 52621, Israel; 2 Functional Genomics unit, Sheba Medical Center, Affiliated to Tel-Aviv University, Tel Hashomer 52621, Israel; 3 Weizmann Institute of Science, PO Box 26, Rehovot 76100, Israel; 4 Institute of Medical Oncology and Radiotherapy, Sheba Medical Center, Affiliated to Tel-Aviv University, Tel Hashomer 52621, Israel; 5 Department of Molecular Genetics, Felsenstein Medical Research Center, Rabin Medical Center, 14 Kaplan street, Petah-Tikva 49100, Israel

**Keywords:** metastasis, WISP-1, DNA microarrays, Gene expression, D122 Lewis lung carcinoma, B16-F10.9 melanoma

## Abstract

Despite advances in the management of solid tumours, the development of metastases continues to be the most significant problem and cause of death for cancer patients. To define genetic determinants of pulmonary metastases, we have applied oligonucleotide microarrays to established murine models of highly metastatic D122 Lewis lung carcinoma and B16-F10.9 melanoma cell lines. These models are characterised by primary subcutaneous growth in C57BL/6J mice, a period of minimal residual disease and spontaneous pulmonary metastases. Microarray analysis defined seven genes, namely – arginase, brain natriuretic peptide (BNP), interleukin-1 alpha (IL-1 alpha), plasminogen activator inhibitor-2 (PAI-2), surfactant protein C (SP-C), uteroglobin (UG) and wnt-1-induced secreted protein-1 (WISP-1), which were consistently elevated in pulmonary metastases compared to the primary tumour of both D122 and B16-F10.9 models. Previous studies demonstrated that two of these seven genes, IL-1 alpha and PAI-2, are involved in the metastatic process. The results obtained by the microarrays were confirmed by real-time quantitative PCR, for three chosen genes – PAI-2, WISP-1 and UG. Our approach aimed to identify genes essential for the metastatic process in general and for pulmonary metastases specifically. Further research should address the precise role of these genes in the metastasising process to the lungs and test if they could be used as targets for future therapies.

A crucial event during cancer progression is the switch from a locally growing tumour to a metastatic one. Tumour development and metastasis are complex processes that include transformation, proliferation, resistance to apoptosis, invasion, neovascularisation and metastatic spread (Liotta *et al*, 1991). Studies to identify molecules involved in tumour development and progression have resulted in the identification of a number of individual candidates that function as cell growth factors (Grandis *et al*, 1998), adhesion molecules (Van Waes *et al*, 1991, 1995), proteases (Andreasen *et al*, 2000), and cytokines that promote inflammation and angiogenesis (Chen *et al*, 1998, 1999).

The discovery of these molecules may be useful in developing new therapeutic interventions. For instance, synthetic Arg-Gly-Asp (RGD) containing peptides can disrupt integrin function (e.g., vitronectin receptor) and successfully inhibit both *in vitro* and *in vivo* melanoma cell invasion (Gehlsen *et al*, 1988).

Metastasis is a multiple-step process in which cancer cells leave the primary tumour site and relocate in a remote organ. This process involves interactions between cancer cells and their surrounding microenvironment. Tumours that metastasise do so to preferred target organs. To explain this apparent specificity, over 100 years ago, Paget formulated his ‘seed and soil’ hypothesis; that is, cells from a given tumour would ‘seed’ only favourable ‘soil’ in a target organ, one that supports colonisation for metastasis to occur.

Several studies used an *in vivo* selection scheme to select highly metastatic cell lines (Clark *et al*, 2000; Chen *et al*, 2001; Khanna *et al*, 2001). By analysing these cells on microarrays, gene expression patterns that correlate with progression to a metastatic phenotype have been defined. Clark *et al* (2000) have shown that the small GTPase RhoC enhances metastasis when overexpressed, whereas a dominant-negative Rho inhibits metastasis. Analysis of the phenotype of cells expressing dominant-negative Rho or RhoC indicates that RhoC is important in tumour cell invasion. The process of selecting highly metastatic cell lines, as done by Clark *et al* (2000), resulted in a large number of genes related solely to metastasis, but not to the microenvironmental changes in the preferred target organs.

To provide insight into mechanisms that allow tumours to metastasise specifically to the lungs, we searched for the genes coordinately overexpressed in pulmonary metastases of two distinct metastatic cell lines of different embryonic origin. The cell lines we used were the highly metastatic mouse D122 Lewis lung carcinoma and B16-F10.9 melanoma. We hypothesised that genes overexpressed in pulmonary metastases of both cell lines, might play a key role in both metastasis and homing of the metastasising cells, specifically to the lungs.

## MATERIALS AND METHODS

### Mice and cell cultures

Ten inbred male and female C57BL/6J mice, 12 weeks old, were grown and maintained in the animal facilities of the Weizmann Institute of Science and Tel-Aviv University. All animal experiments have been carried out with ethical committee approval. The ethical guidelines that were followed meet the standards required by the UKCCCR guidelines (Workman *et al*, 1998).

The highly metastatic D122 clone of the C57BL/6-derived Lewis lung carcinoma (Eisenbach *et al*, 1983) was maintained in DMEM supplemented with 10% heat-inactivated fetal calf serum, glutamine, combined antibiotics, sodium pyruvate and nonessential amino acids. The highly metastatic B16-F10.9 clone of the C57BL/6-derived malignant melanoma (Porgador *et al*, 1989) was grown in DMEM supplemented with 10% heat-inactivated fetal calf serum, glutamine and combined antibiotics.

The mice were divided into four groups. Groups 1 and 2, consisting of three mice each, male and female, respectively, were injected intra-footpad with 2 × 10^5^ D122 or B16-F10.9 cells/mouse, respectively. Groups 3 and 4, consisting of two mice each, male and female, respectively, served as a control. After 5 weeks, when primary tumours reached ∼0.8 cm in diameter, animals were anaesthetized and tumours were dissected and stored at −80°C. After an additional 4 weeks, lungs containing pulmonary metastases were dissected. The lungs of the mice in the control groups were dissected at the matching age. All specimens were snap-frozen in liquid nitrogen.

### Preparation of labelled cRNA

Probe preparation was performed as recommended by the manufacturer of the microarrays (*Affymetrix*, 1998). Briefly, total RNA was isolated from pooled primary tumours and pooled lungs by homogenisation in ice-cold Trizol (Invitrogen). The RNA was then used as template for double-stranded cDNA synthesis with an oligo(dT)_24_ primer containing a T7 RNA polymerase promoter site added to the 3′ end (Genset, La Jolla, CA, USA). The cDNA was extracted with phenol/chloroform, ethanol-precipitated, and used as a template for *in vitro* transcription (Ambion T7 Megascript system) with biotin-labelled nucleotides (Enzo Diagnostics). Labelled cRNA was fragmented and a hybridisation mix was generated as recommended (*Affymetrix*, 1998).

### Hybridisation of microarrays

Aliquots of each sample (10 *μ*g cRNA in 200 *μ*l hybridisation mix) were hybridised to a Genechip Hugene FL array. After hybridisation, each array was washed, stained with streptavidin phycoerythrin (Molecular Probes), washed again, hybridised with biotin labelled antistreptavidin phycoerythrin antibodies, restained with streptavidin phycoerythrin (Molecular Probes), and scanned (Hewlett-Packard, GeneArray scanner G2500A).

### Analysis of Genechip data

Scanned output files were visually inspected for hybridisation artefacts and then analysed using Genechip® 4.0 software (Affymetrix). Arrays were scaled to an average intensity of 600 per gene and analysed independently. The expression value for each gene was determined by calculating the average of differences (perfect match intensity minus mismatch intensity) of the probe pairs in use for this gene. The expression analysis files created by Genechip® 4.0 software were then transferred to a database (Microsoft Access) linked to Internet genome databases (e.g. NHLBI, Swiss Prot and GeneCards) to update gene definitions. A value of 20 was assigned to all measurements lower than 20. We did not include in the analysis genes that did not have at least one average difference intensity value ⩾100 which was defined as ‘present’ call by Affymetrix criteria. For cluster analysis, we used CLUSTER and TREEVIEW programs described by Eisen *et al* (1998). Fold ratios were calculated for each primary tumour against the pulmonary metastases of the same model and for the pulmonary metastases against the normal lung of the same gender. To ensure that the enhanced expression of genes in the pulmonary metastases was not due solely to the influence of the microenvironment, we removed genes with a higher level of expression in the normal murine lung compared to the pulmonary metastases. This criterion filtered 55, 78 and 91% of the genes that were otherwise considered as overexpressed in the D122, B16-F10.9 and both models, respectively.

### Real-time quantitative PCR analysis

Relative quantitative PCR was performed using the LightCycler System® (Roche Diagnostics). An aliquot of 6 *μ*g of the previously extracted total RNA was reverse transcribed using Moloney Murine Leukemia Virus-Reverse Transcriptase (RT) and random hexamers as primer (Promega). The obtained cDNA was diluted 1/10 with water and 5 *μ*l was used for amplification. The PCR was performed with the LightCycler FastStart DNA SYBR GreenI Kit (Roche Diagnostics) according to the protocol provided in the kit. To control for specificity of the amplification products, a melting curve analysis was performed. No amplification of unspecific products was observed. In addition, PCR products were gel separated to confirm a band of the expected size.

The relative initial amount of cDNA of a particular template in the cDNA mixture was extrapolated from a standard curve. The standards, composed of five serial dilutions of one of the cDNAs, ranging from 1/5 to 1/1000 of the original cDNA, were run in parallel with the samples under identical PCR conditions, amplified with the same set of primers. The slope of the standard curve was converted to the amplification efficiency *E* by the following algorithm: *E*=10^−1/^*^slope^*. The primers used for all four genes – PAI-2, RhoC, WISP-1 and UG had an efficiency >1.85 (data not shown). The relative amounts obtained were normalised by the housekeeping gene beta-actin, whose levels of expression were not changed significantly according to the microarrays (data not shown). The detailed procedure for quantification has been described by Ginzinger (2002).

The primer sequences were (5′ to 3′):

beta-actin – CTGAGAGGGAAATCGTGCG, GGTGGTACCACCAGACAAC;

PAI-2 – CTGCTACCCGAAGGTTCTG, GGAAGCAACAGGAGCATGC;

RhoC – GCTGGGCAAGAAGACTACG, CCTTCCTCAGATCGAACCG;

WISP-1 – CGTGGAGCAACGGTATGAG, GAGAGTGAAGTTCGTGGCC;

UG – CATGCTGTCCATCTGCTGC, CTCTTGTGGGAGGGTATCC.

## RESULTS

### Global analysis of gene expression

**Figure 1:**
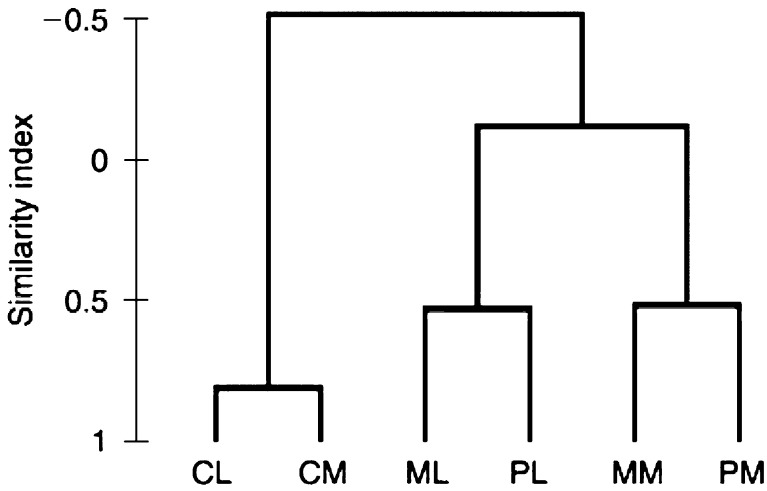
Dendrogram of cluster analysis of microarrays. CL, normal male murine lung – Control for the D122 Lewis lung model; CM, normal female murine lung – control for the B16-F10.9 melanoma model; ML – pulmonary metastases of the D122 Lewis lung model; PL – primary tumour of the D122 Lewis lung model; MM – pulmonary metastases of the B16-F10.9 melanoma model; PM – primary tumour of the B 16-F10.9 melanoma model.

Global analysis of gene expression reveals a correlation between the primary tumour of each model, D122 or B16-F10.9, to its matching pulmonary metastases. In other words, as expected, the diversity between the two models is greater than the diversity between the primary tumour and the pulmonary metastases of the same model. Only genes with at least one average difference intensity value ⩾100, which are defined as ‘present’ by Affymetrix criteria, were included in the cluster analysis (Figure 1

).

### Oligonucleotide microarray analysis

The tumour model described above was used with the highly metastatic mouse D122 Lewis lung carcinoma and B16-F10.9 melanoma cell lines. Microarray experiments examined differences in gene expressions between the subcutaneous primary tumour and the pulmonary metastases. We focus on the genes that were overexpressed in the pulmonary metastases compared to the primary tumour, since we assume that transcripts overexpressed in metastatic tissue have applicable diagnostic and therapeutic potential. Forty-two and 16 genes were overexpressed in pulmonary metastases derived from the mouse D122 and B16-F10.9 cell lines, respectively (Tables 1a and b

**Table 1:** Functional groups of genes that were overexpressed, at a fold ⩾2.5, in pulmonary metastases compared to primary tumour in the (a) D122 model and (b) B16-F10.9 model

**Description**	**GenBank accession #**	**Fold ratio**
(a) *D122 model*		
Apoptosis		
Caspase 3^a^	U54803	2.9
miap-3	U88990	3.0
MIHA	U36842	7.1
		
Cell adhesion		
Integrin alpha V (Cd51)^a^	U14135	2.9
Islr	AB024538	5.2
Lymphocyte antigen 84^a^	Y07519	2.6
		
Cellular matrix		
Actin, alpha 1	M12347	5.5
histone H2a(A)-613, H2a(B)-613 and H2b-613 (H2b)	U62673	4.1
		
Differentiation		
H19	X58196	3.0
Madr2	U60530	2.7
		
ECM/angiogenesis		
Collagen type VI, alpha 3 subunit	AF064749	9.7
Enamelin	U82698	10.1
Fibronectin	M18194	4.5
Matrilin-2 precursor^a^	U69262	4.0
Procollagen type X, alpha 1	X67348	3.8
		
Membrane and nuclear membrane		
YE1/48	M25775	3.1
		
Metabolism		
Acetyl coenzyme A dehydrogenase, long-chain^a^	U21489	2.6
AKR1C13	AB027125	9.7
Ferrochelatase	M61215	3.0
griPGHS^a^	M88242	6.3
Pex	U75646	4.4
		
Receptors/ligands		
Bacteria binding macrophage receptor MARCO	U18424	7.4
GRIP1	U39060	6.4
MIP2	X53798	7.9
		
Signal transduction		
Mess1^a^	U28726	14.5
MPA	X60664	2.5
WSB-2	AF033188	2.6
		
Transcription		
mafF^a^	AB009694	10.9
Slug, chicken homologue	U79550	2.5
zinc finger gene OZF^a^	AJ224763	5.8
		
Translation		
*N*-myristoyltransferase 2^a^	AF043327	10.8
Ribosomal protein (Rpl12)	L04280	2.8
		
Other		
Dnmt2^a^	AF012129	2.8
homer-2a	AF093259	3.7
Lysophospholipase I	U89352	10.4
Modifier 2 protein^a^	X56683	2.5
P35B^a^	X53619	2.6
PLA2	M72394	2.6
Retinaldehyde-binding protein	AF084642	4.9
SCID complementing gene 2^a^	D78188	3.6
Sfrp2	U88567	6.8
Synaptobrevin-like protein	X96737	3.1
		
(b) *B16-F10.9 model*		
Apoptosis		
P53-variant^b^	U59758	3.9
		
ECM/angiogenesis		
Hyaluronidase 1	AF011567	5.8
Minopontin	X13986	8.7
VEGF-C	U73620	4.6
		
Metabolism/proteosome		
beta-TrCP protein E3RS Ikappa B^b^	AF099932	7.9
Ceramide glucosyltransferase	D89866	15.0
		
Signal transduction		
cAMP-dependent Rap 1	AF115480	6.3
LIM and SH3 protein 1	U58882	5.2
RAB7, member RAS oncogene familly	Y13361	2.8
		
Receptors/ligands		
Mouse homologue of human ocular albinism 1	X98352	2.7
		
Transcription/translation		
Cyclin T1^b^	AF095640	3.5
Myeloblastosis oncogene-like 1	L35261	2.9
Zinc-finger protein 40	D10632	5.1
		
Other		
HSP-105	L40406	2.5
Mouse Ig kappa chain V-region	M18237	16.5
Potassium channel alpha subunit^b^	AF008574	2.9

a Indicates genes that were underexpressed in the B16-F10.9 model.

b Indicates genes that were underexpressed in the D122 model. Genes that were overexpressed in both models are not shown.

). Seven genes, namely – arginase, brain natriuretic peptide (BNP), interleukin-1 alpha (IL-1 alpha), plasminogen activator inhibitor-2 (PAI-2), surfactant protein C (SP-C), uteroglobin (UG) and wnt-1-induced secreted protein-1 (WISP-1), were overexpressed in pulmonary metastases of both cell lines (Table 2

**Table 2:** Genes that were overexpressed, at a fold ⩾2.5, in pulmonary metastases compared to primary tumour in both D122 and B16-F10.9 models

	**GenBank accession #**	**Fold ratio**	
**Description**		**D122 model**	**F10.9 model**
Arginase	U51805	22.5	4.9
BNP	D16497	2.7	2.7
IL-1 alpha	M14639	3.1	7.9
PAI-2	X16490	4.4	2.5
SP-C	AA921489	239.3	102.5
UG	L04503	383.6	27.7
WISP-1	AF100777	3.7	4.5

).

**Figure 2:**
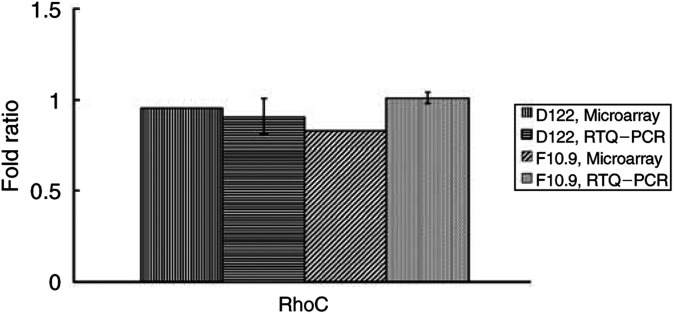
Comparative ratio analysis by RTQ–PCR and microarrays. Fold ratios were calculated for each primary tumour against the pulmonary metastases. RTQ–PCR results (mean±s.d.) were obtained from three independent experiments, each in duplicate.

We could not detect any consistent difference in the expression of RhoC in pulmonary metastases compared to the primary tumour. As this gene has been previously shown to be important in the metastatic phenotype of melanoma cell lines (Clark *et al*, 2000), we confirmed this microarray result using real-time quantitative PCR (RTQ–PCR) (Figure 2

).

### RTQ–PCR confirms overexpression of PAI-2, WISP-1 and UG in pulmonary metastases of both cell lines

**Figure 3:**
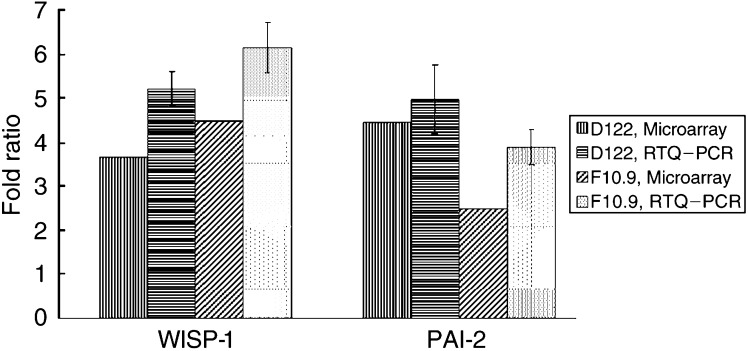
Comparative ratio analysis by RTQ–PCR and microarrays. Fold ratios were calculated for each primary tumour against the pulmonary metastases. RTQ–PCR results (mean±s.d.) for WISP-1 and PAI-2 were obtained from three and four independent experiments, respectively, each in duplicate (PAI-2 results are illustrated by two representative experiments).

To further investigate the reliability of our array data, we measured the expression levels of PAI-2, WISP-1 and UG that were overexpressed in both models using the LightCycler System® (Roche Diagnostics). The expression levels of PAI-2, WISP-1 and UG measured by RTQ–PCR were similar to those measured by the microarrays (Figure 3

, UG data not shown).

## DISCUSSION

Previous studies provided insight into the patterns of gene expression that allow tumours to metastasise by comparing highly metastatic *vs* low metastatic cell lines. This approach identified genes that were overexpressed within the highly metastatic cell line and that were, in general, essential to the nonmetastatic/metastatic switch. One study using this approach demonstrated that the small GTPase RhoC was essential for the enhancement of melanoma metastasis when overexpressed. Furthermore, analysis of the phenotype of melanoma cells expressing dominant-negative Rho or RhoC indicated that RhoC is important in tumour cell invasion (Clark *et al*, 2000).

In this study, we could identify essential targets expressed within the tumour cell, as a result of interactions with the surrounding microenvironment. The reason for this is the comparison made between two phases of a tumour progression *in vivo* – the primary site and the metastases, both with the surrounding tissue, originating from a highly metastatic cell line. Our attitude aimed to emphasise genes that could be essential for metastasis to the lungs and might play a key role in homing the tumour cells specifically to that organ. Therefore, in our study, the small GTPase RhoC was not overexpressed in the pulmonary metastases compared to the primary tumour of both cell lines. We therefore argue that RhoC has general importance in tumour invasiveness and in the nonmetatstatic/metastatic switch, but not in the specific metastatic homing to the lungs.

Previously, Gemma *et al* (2001) compared the expression profiles of two subpopulations of an adenocarcinoma cell line with high metastatic potential – PC9/f9 and PC9/f14 – with the parent cell line PC9 using cDNA array. They have shown that the expression of matrix metalloproteinase-2 (MMP-2), plasminogen activator inhibitor-1 (PAI-1) and IL-1 alpha was overexpressed in the highly metastatic subpopulations. There is already abundant experimental evidence that the plasminogen activator system plays a key role in tumour invasion and metastasis (Andreasen *et al*, 1997; Schmitt *et al*, 1997). A critical balance of uPA, its cell surface receptor uPA-R (CD 87), and its inhibitor PAI-1 is the prerequisite for efficient focal proteolysis, adhesion and migration, and hence, subsequent tumour cell invasion and metastasis. There is evidence that PAI-2 may block uPA-mediated proteolysis and is inversely correlated with tumour progression of nonsmall cell lung carcinomas and neuroendocrine lung tumours (Robert *et al*, 1999) and breast cancer (Borstnar *et al*, 2002). In contrast, higher expression levels of PAI-2 were found in tumour tissues as compared to their normal equivalents and significant relations were found between PAI-2 and pathological stage in nonsmall cell lung cancer patients (Salden *et al*, 2000). Our data suggest a key role for PAI-2 in lung metastasis from different tumour origins.

Wnt-1-induced secreted protein (WISP-1) was identified by Pennica *et al* (1998) as an oncogene regulated by the Wnt-1-beta-catenin pathway and is a member of the CCN family of growth factors, which are cysteine-rich, heparin-binding, secreted proteins associated with the extracellular matrix, and can interact with cellular integrins. Xu *et al* (2000) identified WISP-1 as beta-catenin-regulated and its forced overexpression in normal rat kidney fibroblasts (NRK-49F) was sufficient to induce their transformation. Recently, it was shown that WISP-1 can both activate the anti-apoptotic Akt/PKB signalling pathway and act to block cell death in the p53-mediated apoptosis pathway (Su *et al*, 2002).

Wnt-1-induced secreted protein-1 was overexpressed (2 to >25-fold) in 84% of human colon tumours examined compared with patient-matched normal adjacent mucosa (Pennica *et al*, 1998). The expression levels of WISP-1, in mammary tumours from Wnt-1 transgenic mice, were high in stromal fibroblasts lying within the fibrovascular tumour stroma, but low focally within tumour cells (Pennica *et al*, 1998). Elevated levels of WISP-1 in primary breast cancer were associated with more advanced features, such as tumour size and metastases to axillary lymph nodes (Xie *et al*, 2001).

In contrast, WISP-1 was found to be one of the 70 prognostic reporter genes able to identify clinical outcome in primary breast cancer patients. It was shown that WISP-1 was overexpressed in the good prognosis group of patients, who did not develop distant metastases within 5 years (Van't Veer *et al*, 2002). Furthermore, the mouse WISP-1 is identical to the gene identified as ELM1 (GenBank accession# AB004873). ELM1 is expressed in low, but not high, metastatic mouse melanoma cells, and suppresses the *in vivo* growth and metastatic potential of K-1735 mouse melanoma cells (Hashimoto *et al*, 1998). The contradiction between the above findings may be explained in view of previous studies suggesting that the effect of a single gene may differ according to the genetic and cellular context. For example, c-MYC can induce both cell proliferation and apoptosis (Evan *et al*, 1992; Jain *et al*, 2002; Pelengaris et al, 2002). Another example was given by Serrano *et al* (1997), who showed that the oncogene ras, known for its ability to transform immortal rodent cells to a tumourigenic state, can provoke premature cell senescence in primary human and rodent cells.

Here we have demonstrated overexpression of WISP-1 in pulmonary metastases of two distinct metastatic cell lines. We propose a key role for WISP-1 in the metastasising process to the lungs.

Interleukin-1 alpha was shown to enhance the adhesion of A549 lung carcinoma and M6 melanoma cells to the vascular surface both *in vitro* and *in vivo*, suggesting that IL-1 proteins facilitate the metastatic process (Lauri *et al*, 1990). Significant correlation between expression of IL-1 alpha and liver metastasis in gastric carcinoma was shown (Tomimatsu *et al*, 2001), suggesting that IL-1 alpha functions universally in the metastatic process. Here we have demonstrated overexpression of IL-1 alpha in pulmonary metastases of two distinct metastatic cell lines.

Arginase converts L-arginine to L-ornithine, which is the precursor of polyamines that are essential components of cell proliferation. *N*-hydroxy-L-arginine (NOHA), a stable intermediate product formed during conversion of L-arginine to NO by nitric oxide synthase, inhibited proliferation of the high arginase-expressing MDA-MB-468 cells, activated caspase-3 and induced apoptosis after 48 h (Singh *et al*, 2000). Additionally, Chang *et al* (2001) have shown that arginase induction in macrophages can enhance tumour cell growth by providing them with polyamines and suppress tumour cytotoxicity by reducing NO production. The arginase inhibitor L-norvaline downregulated this proliferative effect. Yet, an active subunit of arginase was found to inhibit differentially poorly liver metastasizing cell lines (Cavanaugh and Nicolson, 2000). Highly metastatic cells displayed no ability to preferentially inactivate or inhibit the activity of arginase, but they did display slightly greater amounts of intracellular arginine. Here, however, arginase was overexpressed in pulmonary metastases of two distinct metastatic cell lines. Therefore, arginase may be essential for tumour metastasis to the lungs, but not to the liver, and lung macrophages might take part in that process.

Brain natriuretic peptide (BNP) is a polypeptide hormone, demonstrated mainly in the heart, which causes vasodilation and natriuresis in man and animals. Elevated levels of BNP are usually associated with cardiac dysfunction (Cowie and Mendez, 2002). There is also evidence that human small cell lung cancer cell lines produce BNP (Ohsaki *et al*, 1999). There is no known connection of BNP to tumourigenesis or metastasis.

Uteroglobin (UG) was found to be infrequently expressed in non-small cell lung cancer cell lines, despite being abundantly produced by progenitor cells for normal and neoplastic airway epithelium. A549 cells transfected with UG demonstrated a marked reduction in invasiveness and decreased adhesiveness to fibronectin compared with the controls (Szabo *et al*, 1998). Surfactant-C (SP-C) is overexpressed in normal lungs and plays an important role in its functioning. Our data show overexpression of UG and SP-C in pulmonary metastases compared to both primary tumours and normal lung. Nevertheless, these genes were also overexpressed in the normal lung compared to the primary tumour.

Most of the previously described genetic alterations in various cancers involve inactivation of tumour suppressor genes. The proteins produced from these genes are difficult to target with drugs, because they are inactive or absent in the cancer cells. In contrast, enzymes whose expression is elevated in cancer cells like those encoded by WISP-1, arginase, IL-1 alpha and PAI-2 provide excellent targets for drug discovery purposes. Further research should address the specific role of these genes in the formation of pulmonary metastases from different tumours.

## References

[bib1] Affymetrix (1998) Eukaryotic Expression Analysis Target Preparation. Santa Clara, CA: Affymetrix

[bib2] Andreasen PA, Egelund R, Petersen HH (2000) The plasminogen activation system in tumor growth, invasion, and metastasis. Cell Mol Life Sci 57: 25–40 10949579 10.1007/s000180050497 PMC11146824

[bib3] Andreasen PA, Kjöller L, Christensen L, Duffy MJ (1997) The urokinase-type plasminogen activator system in cancer metastasis: a review. Int J Cancer 72: 1–22 9212216 10.1002/(sici)1097-0215(19970703)72:1<1::aid-ijc1>3.0.co;2-z

[bib4] Borstnar S, Vrhovec I, Svetic B, Cufer T (2002) Prognostic value of the urokinase-type plasminogen activator, and its inhibitors and receptor in breast cancer patients. Clin Breast Cancer 3: 138–146 12123538 10.3816/CBC.2002.n.018

[bib5] Cavanaugh PG, Nicolson GL (2000) Partial purification of a liver-derived tumor cell growth inhibitor that differentially inhibits poorly-liver metastasizing cell lines: identification as an active subunit of arginase. Clin Exp Metastasis 18: 509–518 11592308 10.1023/a:1011851131504

[bib6] Chang CI, Liao JC, Kuo L (2001) Macrophage arginase promotes tumor cell growth and suppresses nitric oxide-mediated tumor cytotoxicity. Cancer Res 61: 1100–1106 11221839

[bib7] Chen JJ, Peck K, Hong TM, Yang SC, Sher YP, Shih JY, Wu R, Cheng JL, Roffler SR, Wu CW, Yang PC (2001) Global analysis of gene expression in invasion by a lung cancer model. Cancer Res 61: 5223–5230 11431363

[bib8] Chen Z, Colon I, Ortiz N, Callister M, Dong G, Pegram MY, Arosarena O, Strome S, Van Waes C (1998) Effects of IL-1*α*, IL-1RA and neutralizing antibody on proinflammatory cytokine expression by human squamous cell carcinoma lines. Cancer Res 58: 3668–3676 9721877

[bib9] Chen Z, Malhotra PS, Thomas GR, Ondrey FG, Duffey DC, Enamorada I., Mousa S, Van Waes C (1999) Expression of proinflammatory and proangiogenic cytokines in human head and neck patients. Clin Cancer Res 5: 1369–1379 10389921

[bib10] Clark EA, Golub TR, Lander ES, Hynes RO (2000) Genomic analysis of metastasis reveals an essential role for RhoC. Nature 406: 532–535 10952316 10.1038/35020106

[bib11] Cowie MR, Mendez GF (2002) BNP and congestive heart failure. Prog Cardiovasc Dis 44: 293–321 12007084 10.1053/pcad.2002.24599

[bib12] Eisen MB, Spellman PT, Brown PO, Botstein D (1998) Cluster analysis and display of genome-wide expression patterns. Proc Natl Acad Sci USA 95: 14863–14868 9843981 10.1073/pnas.95.25.14863 PMC24541

[bib13] Eisenbach L, Segal S, Feldman M (1983) MHC imbalance and metastatic spread in Lewis lung carcinoma clones. Int J Cancer 32: 113–120 6862690 10.1002/ijc.2910320118

[bib14] Evan GI, Wyllie AH, Gilbert CS, Littlewood TD, Land H, Brooks M, Waters CM, Penn LZ, Hancock DC (1992) Induction of apoptosis in fibroblasts by c-myc protein. Cell 69: 119–128 1555236 10.1016/0092-8674(92)90123-t

[bib15] Gehlsen KR, Argraves WS, Piersbacher MD, Ruoslahti E (1988) Inhibition of *in vitro* cell invasion by Arg-Gly-Asp-containing peptides. J Cell Biol 106: 925–930 2450101 10.1083/jcb.106.3.925 PMC2115099

[bib16] Gemma A, Takenaka K, Hosoya Y, Matuda K, Seike M, Kurimoto F, Ono Y, Uematsu K, Takeda Y, Hibino S, Yoshimura A, Shibuya M, Kudoh S (2001) Altered expression of several genes in highly metastatic subpopulations of a human pulmonary adenocarcinoma cell line. Eur J Cancer 37: 1554–1561 11506965 10.1016/s0959-8049(01)00154-x

[bib17] Ginzinger D (2002) Gene quantification using real-time quantitative PCR: an emerging technology hits the mainstream. Exp Hematol 30: 503–512 12063017 10.1016/s0301-472x(02)00806-8

[bib18] Grandis JR, Melhem MF, Gooding WE, Day R, Holst VA, Wagener MM, Drenning SD, Tweardy DJ (1998) Levels of TGF-*α* and EGFR protein in head and neck squamous cell carcinoma and patient survival. J Natl Cancer Inst 90: 824–832 9625170 10.1093/jnci/90.11.824

[bib19] Hashimoto Y, Shindo-Okada N, Tani M, Nagamachi Y, Takeuchi K, Shiroishi T, Toma H, Yokota J (1998) Expression of the Elm1 gene, a novel gene of the CCN (connective tissue growth factor, Cyr61/Cef10, and neuroblastoma overexpressed gene) family, suppresses *In vivo* tumor growth and metastasis of K-1735 murine melanoma cells. J Exp Med 187: 289–296 9449709 10.1084/jem.187.3.289 PMC2212122

[bib20] Jain M, Arvanitis C, Chu K, Dewey W, Leonhardt E, Trinh M, Sundberg CD, Bishop JM, Felsher DW (2002) Sustained loss of a neoplastic phenotype by brief inactivation of MYC. Science 297: 102–104 12098700 10.1126/science.1071489

[bib21] Khanna C, Khan J, Nguyen P, Prehn J, Caylor J, Yeung C, Trepel J, Meltzer P, Helman L (2001) Metastasis-associated differences in gene expression in a murine model of osteosarcoma. Cancer Res 61: 3750–3759 11325848

[bib22] Lauri D, Bertomeu MC, Orr FW, Bastida E, Sauder D, Buchanan MR (1990) Interleukin-1 increases tumor cell adhesion to endothelial cells through an RGD dependent mechanism: *in vitro* and *in vivo* studies. Clin Exp Metastasis 8: 27–32 2293911 10.1007/BF00155590

[bib23] Liotta LA, Steeg PS, Stetler-Stevenson WG (1991) Cancer metastasis and angiogenesis: an imbalance of positive and negative regulation. Cell 64: 327–336 1703045 10.1016/0092-8674(91)90642-c

[bib24] Ohsaki Y, Gross AJ, Le PT, Oie H, Johnson BE (1999) Human small cell lung cancer cells produce brain natriuretic peptide. Oncology 56: 155–159 9949302 10.1159/000011957

[bib25] Pelengaris S, Khan M, Evan GI (2002) Suppression of Myc-induced apoptosis in beta cells exposes multiple oncogenic properties of Myc and triggers carcinogenic progression. Cell 109: 321–334 12015982 10.1016/s0092-8674(02)00738-9

[bib26] Pennica D, Swanson TA, Welsh JW, Roy MA, Lawrence DA, Lee J, Brush J, Taneyhill LA, Deuel B, Lew M, Watanabe C, Cohen RL, Melhem MF, Finley GG, Quirke P, Goddard AD, Hillan KJ, Gurney AL, Botstein D, Levine AJ (1998) WISP genes are members of the connective tissue growth factor family that are up-regulated in wnt-1-transformed cells and aberrantly expressed in human colon tumors. Proc Natl Acad Sci USA 95: 14717–14722 9843955 10.1073/pnas.95.25.14717 PMC24515

[bib27] Porgador A, Feldman M, Eisenbach L (1989) H-2Kb transfection of B16 melanoma cells results in reduced tumorigenicity and metastatic competence. J Immunogenet 16: 291–303 2639904 10.1111/j.1744-313x.1989.tb00475.x

[bib28] Robert C, Bolon I, Gazzeri S, Veyrenc S, Brambilla C, Brambilla E (1999) Expression of plasminogen activator inhibitors 1 and 2 in lung cancer and their role in tumor. Clin Cancer Res 5: 2094–2102 10473092

[bib29] Salden M, Splinter TA, Peters HA, Look MP, Timmermans M, van Meerbeeck JP, Foekens JA (2000) The urokinase-type plasminogen activator system in resected non-small-cell lung cancer. Ann Oncol 11: 327–332 10811500 10.1023/a:1008312801800

[bib30] Schmitt M, Harbeck N, Thomssen C, Wilhelm O, Magdolen V, Reuning U, Ulm K, Höfler H, Jänicke F, Graeff H (1997) Clinical impact of the plasminogen activation system in tumor invasion and metastasis: prognostic relevance and target for therapy. Thromb Haemost 78: 285–296 9198168

[bib31] Serrano M, Lin AW, McCurrach ME, Beach D, Lowe SW (1997) Oncogenic ras provokes premature cell senescence associated with accumulation of p53 and p16INK4a. Cell 88: 593–602 9054499 10.1016/s0092-8674(00)81902-9

[bib32] Singh R, Pervin S, Karimi A, Cederbaum S, Chaudhuri G (2000) Arginase activity in human breast cancer cell lines: *N*(omega)-hydroxy-L-arginine selectively inhibits cell proliferation and induces apoptosis in MDA-MB-468 cells. Cancer Res 60: 3305–3312 10866325

[bib33] Su F, Overholtzer M, Besser D, Levine AJ (2002) WISP-1 attenuates p53-mediated apoptosis in response to DNA damage through activation of the Akt kinase. Genes Dev 16: 46–57 11782444 10.1101/gad.942902 PMC155313

[bib34] Szabo E, Goheer A, Witschi H, Linnoila RI (1998) Overexpression of CC10 modifies neoplastic potential in lung cancer cells. Cell Growth Differ 9: 475–485 9663466

[bib35] Tomimatsu S, Ichikura T, Mochizuki H (2001) Significant correlation between expression of interleukin-1 alpha and liver metastasis in gastric carcinoma. Cancer 91: 1272–1276 11283926 10.1002/1097-0142(20010401)91:7<1272::aid-cncr1128>3.0.co;2-z

[bib36] Van Waes C, Kozarsky KF, Warren AB, Kidd L, Paugh D, Liebert M, Carey TE (1991) The A9 antigen associated with aggressive human squamous carcinoma is structurally and functionally similar to the newly defined integrin *α*6 *β*4. Cancer Res 51: 2395–2402 1750876

[bib37] Van Waes C, Surh DM, Chen Z, Rhim JS, Brager R, Roy SB, Kashima H, Wolf GT, Carey TE (1995) Increase in suprabasilar integrin adhesion molecule expression in human epidermal neoplasms accompanies increased proliferation occurring with immortalization and tumor progression. Cancer Res 55: 5434–5444 7585613

[bib38] Van't Veer LJ, Dai H, van de Vijver MJ, He YD, Hart AA, Mao M, Peterse HL, van der Kooy K, Marton MJ, Witteveen AT, Schreiber GJ, Kerkhoven RM, Roberts C, Linsley PS, Bernards R, Friend SH (2002) Gene expression profiling predicts clinical outcome of breast cancer. Nature 415: 530–536 11823860 10.1038/415530a

[bib39] Workman P, Twentyman P, Balkwill F, Balmain A, Chaplin D, Double J, Embleton J, Newell D, Raymond R, S, Stephens T, Wallace J (1998) United Kingdom Co-ordinating Committee on Cancer Research (UKCCCR) Guidelines for the Welfare of Animals in Experimental Neoplasia. Br J Cancer 77: 1–10 10.1038/bjc.1998.1 PMC2151254 9459138

[bib40] Xie D, Nakachi K, Wang H, Elashoff R, Koeffler HP (2001) Elevated levels of connective tissue growth factor, WISP-1, and CYR61 in primary breast cancers associated with more advanced features. Cancer Res 61: 8917–8923 11751417

[bib41] Xu L, Corcoran RB, Welsh JW, Pennica D, Levine AJ (2000) WISP-1 is a Wnt-1- and beta-catenin-responsive oncogene. Genes Dev 14: 585–595 10716946 PMC316421

